# Patient-Derived Xenografts as an Innovative Surrogate Tumor Model for the Investigation of Health Disparities in Triple Negative Breast Cancer

**DOI:** 10.1089/whr.2020.0037

**Published:** 2020-09-24

**Authors:** Margarite D. Matossian, Alexandra A. Giardina, Maryl K. Wright, Steven Elliott, Michelle M. Loch, Khoa Nguyen, Arnold H. Zea, Frank H. Lau, Krzysztof Moroz, Adam I. Riker, Steven D. Jones, Elizabeth C. Martin, Bruce A. Bunnell, Lucio Miele, Bridgette M. Collins-Burow, Matthew E. Burow

**Affiliations:** ^1^ Section of Hematology and Oncology, Department of Medicine, Tulane University School of Medicine, New Orleans, Louisiana, USA.; ^2^ Biospecimen Core Laboratory, Louisiana Cancer Research Center, New Orleans, Louisiana, USA.; ^3^ Section of Hematology and Oncology, Department of Medicine, Louisiana State University Health Sciences Center, School of Medicine, New Orleans, Louisiana, USA.; ^4^ Department of Genetics and Stanley S. Scott Cancer Center, Louisiana Health Sciences Center, New Orleans, Louisiana, USA.; ^5^ Department of Surgery, Louisiana State University Health Sciences Center, School of Medicine, New Orleans, Louisiana, USA.; ^6^ Department of Pathology, Tulane University School of Medicine, New Orleans, Louisiana, USA.; ^7^ Department of Surgery, DeCesaris Cancer Institute, Anne Arundel Medical Center, Luminis Health, Annapolis, Maryland, USA.; ^8^ Department of Surgery, Tulane University School of Medicine, New Orleans, Louisiana, USA.; ^9^ Department of Biological & Agricultural Engineering, Louisiana State University, Baton Rouge, Louisiana, USA.; ^10^ Department of Pharmacology, Tulane University School of Medicine, New Orleans, Louisiana, USA.

**Keywords:** African ancestry, health disparities, extracellular matrix, metastasis, triple-negative breast cancer, patient-derived xenograft

## Abstract

Despite a decline in overall incidence rates for cancer in the past decade, due in part to impressive advancements in both diagnosis and treatment, breast cancer (BC) remains the leading cause of cancer-related deaths in women. BC alone accounts for ∼30% of all new cancer diagnoses in women worldwide. Triple-negative BC (TNBC), defined as having no expression of the estrogen or progesterone receptors and no amplification of the HER2 receptor, is a subtype of BC that does not benefit from the use of estrogen receptor-targeting or HER2-targeting therapies. Differences in socioeconomic factors and cell intrinsic and extrinsic characteristics have been demonstrated in Black and White TNBC patient tumors. The emergence of patient-derived xenograft (PDX) models as a surrogate, translational, and functional representation of the patient with TNBC has led to the advances in drug discovery and testing of novel targeted approaches and combination therapies. However, current established TNBC PDX models fail to represent the diverse patient population and, most importantly, the specific ethnic patient populations that have higher rates of incidence and mortality. The primary aim of this review is to emphasize the importance of using clinically relevant translatable tumor models that reflect TNBC human tumor biology and heterogeneity in high-risk patient populations. The focus is to highlight the complexity of BC as it specifically relates to the management of TNBC in Black women. We discuss the importance of utilizing PDX models to study the extracellular matrix (ECM), and the distinct differences in ECM composition and biophysical properties in Black and White women. Finally, we demonstrate the crucial importance of PDX models toward novel drug discovery in this patient population.

## Introduction

Breast cancer (BC) remains a global issue despite impressive advances in therapeutic strategies, accounting for 30% of all new cancer diagnoses in women.^1^ Overall, BC remains the most frequently diagnosed cancer worldwide and is the leading cause of cancer-related death.^2^ BC is broadly categorized into three subtypes based on receptors that are expressed or amplified.

Estrogen receptor (ER) and progesterone receptor (PR) positive and HER2/Neu receptor amplified subtypes can be treated with endocrine-targeted or HER2/Neu-targeted therapies, respectively. Triple negative BC (TNBC), a subtype that lacks ER and PR expression and HER2/Neu amplification, is clinically aggressive with high rates of metastasis, chemoresistance, recurrence, and develops in women at a younger age.^3^ TNBC mortality is more pronounced in patients of African and Hispanic ancestries, suggesting specific contributing factors within these patients cohorts contributes to overall survival and prognoses.^4–8^ Socioeconomic factors (low income and poor access to health care) in these patient cohorts significantly affects TNBC incidence and mortality outcomes in Black and Hispanic populations, more so than in non-Black and non-Hispanic populations, referenced as White populations throughout this review.

Preclinical and clinical studies have discovered inherent risk factors and oncogenic pathways upregulated in Black TNBC,^8,9^ providing evidence that cell intrinsic factors contribute to differences in TNBC presentation and drug response. Specific regional variations of TNBC mortality is also evident: mortality is highest in less developed countries such as Fiji, the Bahamas, Nigeria (and other Middle African countries), Macedonia, and Pakistan, whereas more developed countries such as North America, Mexico, and Eastern Asia have significantly lower rates.^10^ BC mortality rates in sub-Saharan Africa, especially Nigeria, are ranked the highest globally.^11,12^

Alarmingly, BC incidence rates have been rising in transitioning countries that had historically low rates; projections for 2035 indicate that less-developed regions will have an increase of new cancer cases by up to 144%, compared with 54% in more developed areas.^13^ In addition to TNBC mortality, overall metastatic BC and TNBC incidence rates also differ. More non-Hispanic Black patients were diagnosed with metastatic cancer compared with other ethnicities (8% and 5%–6%, respectively).^14^ With respect to TNBC incidence, multiple studies have identified higher frequency of TNBC in Black women compared with cohorts with White patients.^15,16^ Better representation of Black patients in TNBC drug discovery research is crucial to understanding the biology of this disease in the population it most affects.

Promising new targeted therapies and drug regimens in BC have emerged in recent years due to impressive advancements in target discovery and translational approaches to assess oncology drug effects. Preclinical model systems that accurately recapitulate human tumor biology to test oncology drug therapies facilitates direct translation of the findings into the clinical setting.^17,18^ Patient-derived xenograft (PDX) models are currently the most accurate models to mimic the microanatomy of human solid tumors *in vivo* (using implanted tumors) and *in vitro* (using patient-derived organoids [PDO] and tumor explants).

The use of PDX models in preclinical studies has vastly improved basic and clinical study outcomes in a variety of solid cancer types, including BC.^19,20^ Because most TNBC-related research and knowledge has been acquired from patients who self-identify as White, this research does not reflect the patient population that is most affected by TNBC: patients of African and Hispanic ancestry. In this study, we discuss the current use of PDX models in TNBC drug discovery research, highlight efforts from various institutions to develop PDX models that represent TNBC patients with diverse ethnicities, and the application of ethnically diverse PDX models to identify cell intrinsic and extrinsic signaling pathways unique to ethnic cohorts.

## Use of PDX Models to Understand the Complexity of BC, Particularly for Ethnic Disparities

Before the emergence of PDX models, the standard research model for solid tumors was immortalized, established cell lines and orthotopic xenografts.^21^ Although these models provided invaluable knowledge regarding cancer biology and drug effects on cellular systems, they were limited in the inability to re-create patient-specific features of tumors. More specifically, these models cannot accurately reflect the tumor architecture, defined as the three-dimensional structure and alignment of tumor matrix, and surrounding stroma and cannot reproduce the cellular heterogeneity that is present in the original patient tumor.^22–24^ Passaging of immortalized cell lines that have been growing in cell culture for years results in the introduction of irreversible alterations in genetic information and behavioral characteristics that were not present in the original tumor.^25^

Using physiologically accurate models in cancer research is crucial to investigating previously unrecognized targets and mechanisms of neoplastic diseases. A translational model system that was introduced in cancer research a few years ago and is now extensively utilized in cancer research are PDX models. These models facilitate direct translation of laboratory discoveries and findings to clinical practice; the reverse is also true, in that these models facilitate translation of clinical observation into therapeutic discovery in the laboratory setting.^20,26^ PDX models are imperative in cancer research efforts as they allow for testing of drugs or drug combinations before testing in actual patients.^27^ PDX models are especially important when studying malignancies that exhibit a complex tumor heterogeneity, including TNBC tumors,^28^ both in cellular composition and cell extrinsic properties due to cancer-protective properties of the tumor microenvironment.^28^ TNBC tumors exhibit robust heterogeneity^29–31^ and each established PDX model provides an opportunity to better understand TNBC biologic properties.

The introduction of PDO, and organoids established from PDX tumors serially transplanted in murine models (PDX-O) in drug development focused research studies provides critical translational links between *in vitro* culture systems and *in vivo* observations.^32^ Organoid cultures preserve much of the complex and unique microenvironment as well as the cellular composition of individual patient tumors.^32–34^ These models are crucial because although tumor explants derived from patients are the most translational model, they cannot be indefinitely maintained in cell culture conditions for long-term experiments. Conversely, organoids preserve the microanatomy of the tumors and can be maintained in culture under “low-attachment” conditions. This is especially important when evaluating drug interactions with the various components that comprise a tumor, such as fibroblasts, extracellular protein composition and structure, tumor cells, and immune components.

**FIG. 1.**
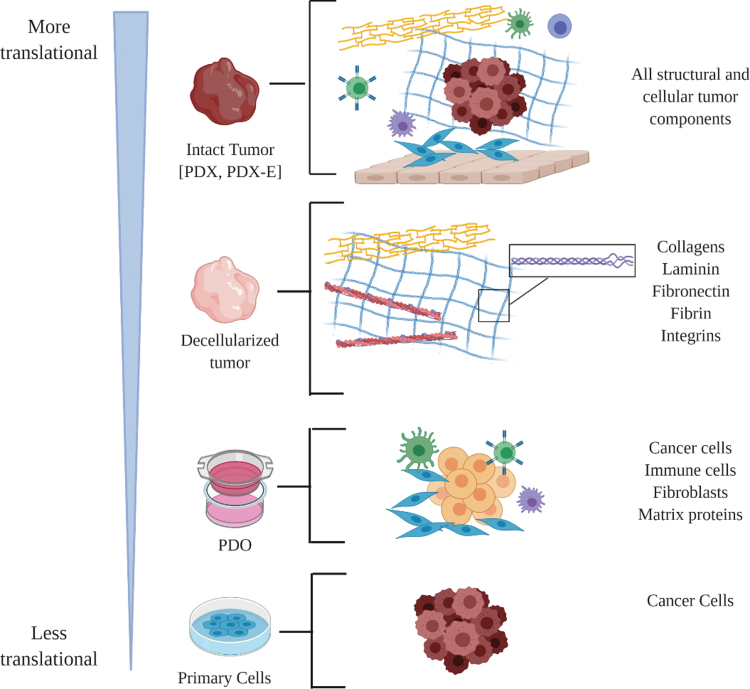
Diverse applications of TNBC PDX models to study various aspects of TNBC biology. Based on the derivation of the PDX model used, different aspects of solid tumors can be analyzed. Examples of model derivations include primary cells isolated from the tumors, PDO that preserve various cell types from the primary tumor, the decellularized tumor to study drug effects on tumor structural components, and the intact tumor either *ex vivo* or *in vivo* (PDX-E). Schematic created with BioRender website. PDO, patient-derived organoids; PDX, patient-derived xenograft; PDX-E, PDX-explants; TNBC, triple-negative breast cancer.

Intact tumor pieces can be treated in a dish, to mimic treating patient tumors in the clinical setting. However, this approach can only be used when sufficient amounts of PDX tumors are available. PDX-derived organoids can be generated from smaller pieces of tissue and expanded. PDX-Os and PDOs have become valuable translational tools to assess drug responses on a larger scale. Incorporating PDX-O and PDO models in cancer research has become an integral part of this era of discovering personalized therapies for individual patients.^34^ A schematic demonstrating how PDX models can be utilized to assess various aspects of TNBC biology is outlined in Figure 1.

## Utilizing TNBC PDX Models to Identify Genes and Signaling Pathways in Ethnic Variants

Unique cell intrinsic molecular and gene signatures have been described in TNBC patients of African ancestry compared with other ethnicities.^35–37^ These findings support the hypothesis that ethnicity and TNBC-specific biomarkers exist for early cancer detection and therapeutic sensitivities.^38,39^ For example, using single-cell gene approaches, Azizi et al. observed significant differences in gene signatures of BC PDX samples derived from Black and White patients.^40^ These included EMT-associated genes (Vimentin, EpCAM, HER2, CDH1, CDH2, TGFβ1, cytokeratins, GATA3, MKI67), CSC markers (ALDH1a1, ALDH1a3, CD44, CD24, CD133), and other genes (YAP1, TM4SF1, TSPAN6, AMOTL2, STAP2, ANXA3). Another approach to identify specific gene or signaling pathways that can be targeted is taking advantage of environmental factors that have higher incidence in Black patients, including obesity.^36,41^ Obesity activates tissue inflammatory responses, activating cancer cell survival, proliferation, and metastasis,^41^ which introduces a plethora of potential targets in TNBC research.^42–45^ Because activation of this pathway has higher incidence in Black patients, agents targeting the obesity/inflammation axis can be a potential strategy to treat certain patients in this cohort. The introduction of PDX models into preclinical drug testing facilitated a translational tool to evaluate single and combination drug therapies in the laboratory setting to test these candidate targets, mimicking clinical trials.^18^

However, the implications of testing candidate targetable genes and signaling pathways in TNBC tumors representing patients of African ancestry in the clinical setting are limited by the number of PDX models available that represent Black and Hispanic patients.^17,18,46^ Of all BC subtypes, TNBC tumors are well represented in PDX models, as demonstrated by Dobrolecki et al. who found that 56% of all breast PDX models (506 patients total) represented TNBC patients.^20^ However, there remains a disparity in the overall representation and understanding of non-European patients among these models. There is an apparent under-representation of African and Black patients with TNBC, highlighting the need to focus upon this higher risk population.

Current therapeutic discovery-focused TNBC research does not adequately address the knowledge gap regarding ethnic disparity in TNBC incidence/mortality rates and biology. To date, most TNBC-related research and knowledge has been acquired from White patients, even though patients with African and Hispanic ancestries represent the majority of TNBC cases. Several institutions in the United States with established biobanks show similar findings with only a fraction of TNBC PDX models that represent Black patients.^46^ The PDXFinder and PDMR public databases were accessed to evaluate racial/ethnic patterns in available PDX models that represent TNBC patients. Out of the 16 total BC models in the PDMR database, 15 had self-reported race data and of those cases 3 represented Black patients and 12 represented White patients. Out of the four specified TNBC PDX models in the PDMR BC database, all were from self-reported White non-Hispanic patients who were European by inferred ancestry. The PDMR site also has 10 2D/3D BC cultures available, and 3 out of 10 models were from self-reported Black or African American patients.^47^ Out of the 104 invasive BC models in the PDXFinder site, 49 had race/ethnicity data available and of those models, 9 were Black patients and 20 were White. Because TNBC is often categorized as the basal-like BC subtype, we accessed basal-like BC samples in the PDXFinder database. Out of the 11 uploaded cases, 5 patients were Black and 6 were White. It is important to note that not all TNBC PDX models from institutions are uploaded to the public databases, and many of the uploaded model information does not specify race/ethnicity of some patients. Furthermore, uploaded PDX models were not characterized by TNBC subtypes, but rather “invasive” or “basal-like.” However, these findings demonstrate an overall disparity in the number of invasive or basal-like BC models representing Black patients. To acquire a more comprehensive understanding of TNBC biology and to evaluate the efficacy of novel therapeutic strategies, we emphasize the importance and necessity of incorporating cohorts of Black patients and specifically patients with African ancestry in TNBC research. Part of addressing this need is to continue to develop a comprehensive and high-content network of tissue specimens from Black TNBC patients. Importantly, these models must be shared among intrainstitutional laboratories, as well as among different institutions, in a collaborative effort. Incorporation of academic institution's research strengths is necessary to address the common goal of characterizing unique gene signatures and the tumor biology of TNBC. We have an important opportunity to pool resources and utilize samples from cancer centers across the United States to explore novel therapeutic options and discover novel targets that are more representative of the population from which the majority of TNBC-afflicted cases are found.

Notably, several groups throughout the United States that are attempting to address this knowledge gap. In 2017, the University of Michigan initiated efforts to establish PDX tumors representative of TNBC patients with African ancestry.^38,46^ Recently, they have expanded their registry to include the Sisters Network in Houston, TX, to recruit more BC patients, with a long-term goal to study germline BC risk in women with diverse racial and ethnic backgrounds in the United States.^48^ At the University of Illinois, Chicago (UIC), there exist similar efforts.^49^ In the Southern United States, MD Anderson Cancer Centers (MDACC), a total of 46/49 of their BC models collected represented patients with TNBC with 19 of these samples procured from Black patients. These programs, in addition to other groups not mentioned, have demonstrated impressive advancements in addressing the limited number of available TNBC PDX models.

**FIG. 2.**
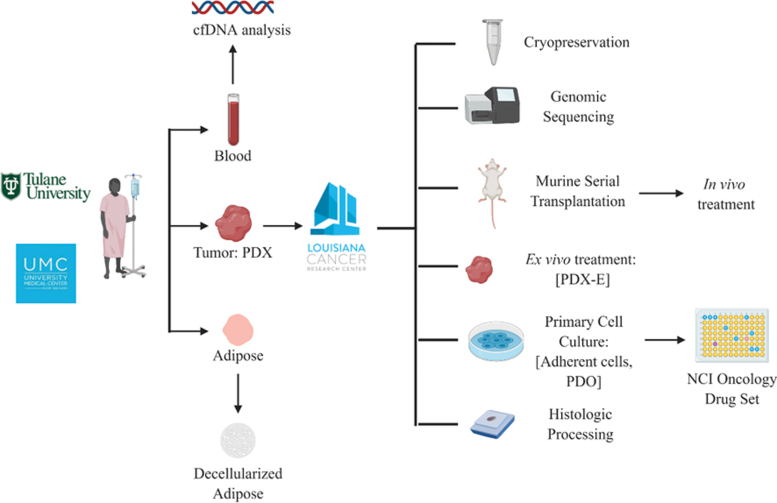
Schematic demonstrating how TNBC PDX tumors that represent Black patients are established and developed from the hospital to laboratory settings. Furthermore, examples of how to potentially use various iterations of PDX tumors to assess ethnic variations of TNBC biology. The NCI oncology drug set contains clinically approved systemic and targeted drugs to test. Schematic created with BioRender website. NCI, National Cancer Institute.

However, to draw more accurate conclusions of ethnic variation and to distinguish ethnic variation from interindividual heterogeneity, hundreds of more representative models are required. Importantly, there exists a paucity of TNBC PDXs that represent Black patients in locations that have high populations of this patient cohort. For example, New Orleans has one of the highest incidence rates of TNBC in the United States, claiming the highest rates in 2016.^50^ By further developing TNBC PDX models from patients in these under-represented communities, we can further address how to optimize treatment regimens for patients representing a variety of ethnicities in TNBC biology (Fig. 2).

## Application of TNBC PDX Models to Address Drug Resistance

Overall, BC (all subtypes) derived from Black patients exhibit a higher degree of intratumoral genetic heterogeneity and more basal-like gene expressions.^51^ Acquisition of drug resistance is one of the defining features of TNBC.^52^ Apoptosis resistance is another contributing factor to BC mortality; important differences in downstream apoptosis regulating genes has been found to be differentially upregulated in tumors representing Black patients compared with respective White patient cohorts.^9^ Although TNBC tumors have a high initial response to chemotherapy, patients with residual disease after completing neoadjuvant chemotherapy have a worse prognosis compared with non-TNBC patients.^52^ Owing to the higher incidence of TNBC in Black populations, and the fact that TNBC tumors have high rates of drug resistance, it is warranted to further investigate the association between African ancestry and acquisition of drug resistance in this tumor type, using translational PDX models.

One potential application of PDX models in TNBC research is to compare treatment-naive tumors with matched post-treatment tumors. These paired tumor tissues are quite valuable, providing insight into the complex changes that occur at the tumor level. Using human tumors from the same patient pre- and post-BC treatment facilitates interrogation of not only cell intrinsic but also cell extrinsic pathways that have been altered in response to drug exposure. Because the extracellular matrix (ECM) of tumors changes in response to drug exposure,^53–57^ investigating how these changes drive drug resistance is important to interrogate, and using human tumors that represent individual tumor's ECM is crucial to these discoveries. There is also value in studying TNBC tumors from patients who have undergone a full course of neoadjuvant chemotherapy, only to have a minimal response to treatment with minimal tumor regression or shrinkage of the primary tumor. These experiments examine possible mechanisms of drug resistance in these patients with TNBC, further highlighted by our ability to compare, in some instances, the specific alterations in gene expression after neoadjuvant chemotherapy.

## Using PDX Models to Understand the Role of the ECM in TNBC Biology of Tumors That Represent Black Patients

TNBC cells depend on the tumor microenvironment, defined as the ECM and other cellular components (immune modulatory cells and fibroblasts) surrounding the cancer cells within a tumor, for survival and tumor progression.^48,49,58^ Comprehensive dissection and examination of the intact tumor in the laboratory setting is crucial to examine the interplay of complex cell–cell and cell–stromal interactions.^59^ PDX models are one of the most accurate systems, to date, for assessing the tumor's unique matrix composition and cellular interactions within the ECM.^60,61^ Although cancer-on-a-chip models can be used to study distinct cell–stroma–microenvironment interactions, they do not truly reflect the intact original patient tumor.^62^ Furthermore, treatment of PDX models provides more accurate data when compared with cell-line-derived xenografts, cell line-based experiments, and other *ex vivo* models.^63^ Targeting the ECM is emerging as a novel therapeutic approach in invasive cancers and is especially important in cancer subtypes that do not have commonly targetable receptors, such as with TNBC.^64–66^ Schedin and Borges discovered important links between breast tissue development and the tumor microenvironment.^67–69^ They found that breast tissue involution after lactation resulted in tissue inflammation and wound healing pathways that deposited high-risk crosslinked fibrillar collagen, which has been associated with poor survival in European American women with invasive BC.^69,70^ These studies demonstrate the importance of the ECM in breast development and provides support for additional research into these interactions to improve the current understanding of the ECM's role in regulating BC development.

Differences in the tumor microenvironment composition, specifically pertaining to the ECM, exist in Black compared with White women.^71,72^ For example, mouse mammary glands humanized fibroblasts derived from premenopausal White patients compared with mammary glands humanized with fibroblasts harvested from Black patients resulted in differential expression of ECM-regulated gene pathways.^72^ Aside from ECM-regulated pathways, other microenvironment components tested (tumorigenicity, metastatic behavior, and protease activity) differed in the two tested groups.^73^ Another study found biologic processes related to chemotaxis, angiogenesis, endoplasmic reticulum function, and cell cycle control were differentially expressed based on race and ethnicity in BCs.^73^ Microvessel density and macrophage infiltration were higher in self-identified AA tumors,^73^ further supporting that ethnic variation contributes to distinct changes in the tumor microenvironment.

Another important feature of tumors that can be studied using PDX models is the three-dimensional tissue architecture, as it is unique to individual patients. The tissue architecture, specifically the alignment/orientation biophysical and structural properties of tumors as well as the composition of the ECM fibers within the tumor, has a powerful and influential role in facilitating tumor growth rates and their propensity to metastasize.^74–76^ The mechanics of the heterogeneous matrix directs tumor progression and cell interactions.^77^ In cancer subtypes with heterogeneous cellular components and clinical presentations, such as TNBC, studying the ECM in-depth is critical for the discovery of novel therapeutic strategies. Distinct differences in ECM alignment and organization have been demonstrated in skin of Black and White patients, suggesting unique properties of the ECM structure in relation to patients of different ethnic backgrounds. Disruption of the elastic fiber arrangement and reduced collagen organization were detrimental to the biochemical properties in aged skin of Black persons.^78^ In addition, biomechanical behaviors have also demonstrated the ability to predict tumor cell invasiveness and metastatic potential. From this, it may be inferred that collagen and elastic fiber organization are important in cancer development, with composition of these tumor microenvironment aspects differing between Black and White populations. Using PDX models, specifically whole and intact PDX tumors from primary tissue specimens, preserves the unique ECM fiber alignment and mechanosensing biomechanical properties within human breast tumors.

Overall, ethnic variations may exist in these cell extrinsic tumor characteristics, but the current models utilized to study these tumor properties utilize synthetic or artificial three-dimensional matrix platforms, which do not mimic native breast tissue.^79^ Furthermore, employing the novel technique of tissue decellularization, or removing the cellular background of tumors while preserving the ECM, facilitates examination of tumor architecture and ECM integrity in patient tumors representing various patient ethnicities.^80^ Applying PDX models in these settings is integral, as it represents the only model in which the true representation of individual patients' ECM architecture and composition can be preserved in the laboratory setting.

## Distinguishing Interindividual from Ethnic Variability in PDX-Based Oncologic Research

The utilization of PDX models in oncologic drug discovery research introduced possible confounding factors into studies evaluating the role of ethnicity in tumor biology. Because PDX models accurately represent features of human tumors in the laboratory setting, these models also maintain the features of tumors that are unique to individuals. This presents the possibility that data demonstrating unique expression of gene/molecular/protein expression found in patients representing diverse ethnicities are due to interindividual variation, and not ethnic variation. Distinguishing between interindividual and ethnic variation is important in oncology research, especially in testing drug response and pharmacokinetic studies.^81–85^ Examining biomarker expression in larger cohorts of patients, or using meta-analyses, can help reduce the risk of this confounding effect.^81,86^ As one study concluded, thousands of samples are required to accurately contribute gene lists for predicting outcome in cancer.^87^ Inadequate estimation of interindividual variation leads to inclusion of nonstatistically significant genes.^88^

Research that identifies “gene signatures” in ethnic cohorts and molecular tumor subtypes examine large numbers of representative tumors to reduce the risk of the findings being contributed to interindividual variation. Population studies not only uncover interindividual heterogeneity but also reveal gene signatures that are longitudinally stable within individuals.^89^ Attempts through computational analyses and algorithms have been made to address the role of interindividual variation in population studies.^84,86–88^ We propose that these analyses must be applied in studies examining ethnic variation in TNBC. Given the limitation of TNBC PDX models that represent ethnic patients, more established models are required to draw accurate conclusions with respect to ethnic variation in TNBC.

## Conclusions

Overall, TNBC has higher rates of mortality and poorer prognoses in Black patients, specifically patients of African ancestries. The purpose of this review is to discuss the diverse applications of PDX models in TNBC biology research to identify ethnic variations in this disease. There have been impressive efforts to address the limited number of established TNBC PDX models that represent Black patients to more comprehensively understand TNBC biology by studying patients who are most afflicted by the disease. However, additional models are required to identify biomarkers, gene and molecular signatures, and biophysical properties of TNBC tumors that are unique to patients who represent specific ethnicities. Having a large database of samples is crucial to assess which candidate gene and signaling pathways are due to ethnic variation, and not due to interindividual variation. These efforts will lead to multiple areas of drug discovery and novel therapeutic approaches, providing valuable insight and understanding of the genomic and phenotypic differences for this difficult-to-treat population.

## Abbreviations Used:

BC: breast cancer
ECM: extracellular matrix
ER: estrogen receptor
MDACC: MD Anderson Cancer Centers
PDO: patient-derived organoids
PDX: patient-derived xenograft
TME: tumor microenvironment
TNBC: triple-negative breast cancer
UIC: University of Illinois, Chicago
